# Missing diversity in brain tumor trials

**DOI:** 10.1093/noajnl/vdaa059

**Published:** 2020-05-13

**Authors:** Birra Taha, Graham Winston, Umberto Tosi, Benjamin Hartley, Caitlin Hoffman, Nadia Dahmane, Christopher E Mason, Jeffrey P Greenfield

**Affiliations:** 1 Department of Neurosurgery, University of Minnesota, Minneapolis, MN, USA; 2 Department of Neurological Surgery, New York Presbyterian Hospital, Weill Cornell Medical College, New York, NY, USA; 3 Department of Physiology and Biophysics and Institute for Computational Biomedicine, Weill Cornell Medical College, New York, NY, USA; 4 Feil Family Brain and Mind Research Institute, New York, NY, USA

**Keywords:** clinical trials, diversity, neuro-oncology, New York City, representation

## Abstract

**Background:**

Clinical trials for brain tumors represent a significant opportunity for both patients and providers to understand and combat a disease with substantial morbidity. The aim of this study was to quantify and map ethnic and racial representation in brain tumor trials and examine the potential gaps in trial recruitment. We also show that these representation gaps persist even in large multicultural cities like New York City.

**Methods:**

We analyzed brain tumor clinical trials registered on www.clinicaltrials.gov between July 1, 2005 and completed on or before November 11, 2017. We used a combination of PubMed/MEDLINE and Google Scholar to find associated publications and obtained trial information as well as patient demographic information (when available) including race or ancestry.

**Results:**

Out of 471 trials, 27% had no published results. Only 28.4% of trials with results reported race or ethnicity of trial participants, with no observed upward trend by year. Whites were significantly overrepresented in trials for metastatic brain tumors (*P* < .001) and high-grade trials (*P* < .001). Blacks/African Americans (AAs), Hispanics, and Asians were significantly underrepresented (*P* < .001) in high-grade trials, while only Blacks/AAs were underrepresented in trials for metastatic brain tumors (*P* < .001). Representation gaps were not observed in pediatric trials. Despite being a multicultural hub, New York City displayed similar gaps in trial representation.

**Conclusions:**

Despite increasing representation in the American population, minorities are underrepresented in brain tumor trials. In addition, despite numerous legal requirements and ethical mandates, published results including race-based information are remarkably absent from 70% of brain tumor trials.

**Key Points**

- Despite the potent scientific value of brain tumor clinical trials, 27% of trials had no published results; only 28% of those trials with results reported race information.
- Brain tumor trials contain significant gaps in representation and persist even in dense multicultural centers like New York City.

**Importance of the Study**

Clinical trials for brain tumors represent a significant opportunity for both patients and providers to understand and combat a disease with substantial morbidity. With a rising overall population of non-White patients in the West, and with comparable rates of incidence, attention should be paid toward adequate reporting and recruitment in clinical trials.^1,2^ Ensuring a diverse representation of races/ancestries for brain tumor trials is especially important given the allelic diversity in clinically relevant genes and has implications for most other diseases as well.

Brain tumors represent approximately 1% of all new cancer diagnoses and 2% of yearly cancer deaths.^3^ The distribution and incidence of various brain tumors varies by race, suggesting a potentially important genetic, heritable component in brain tumor pathophysiology. African Americans (AAs) are 4 times more likely to have benign brain tumors (ie, WHO grade I) than malignant (ie, WHO grade III or IV) tumors.^1^ Benign meningiomas are the most common primary brain tumors and their incidence is the highest in AA populations.^2^ Additionally, the incidence of pituitary tumors is higher in AAs than in Caucasian Americans.^4^ Malignant tumors, on the other hand, are most common in Caucasian Americans, with gliomas being the most prevalent subtype. These epidemiological differences among the races necessitate the understanding at the biological/genetic level. Proper planning and reporting of race in clinical trials is needed to account for differences in presentation, treatment, and response.

Historically, ethnic minorities in the United States have been underrepresented in clinical trials.^5^ While this underrepresentation may have improved over time in some cases, outcomes of brain neoplasms clinical trials are rarely stratified by race. A notable exception is glioblastoma (GBM), where an explicit consensus on the effect of race and clinical outcomes has not been reached.^6^ Disagreement between conclusions by different large clinical databases has contributed to this uncertainty.^7,8^ This may be in part due to the dismal prognosis of the disease. Additional studies on the current status of GBM clinical trials to elucidate reasons for these differences are, however, still ongoing.^9,10^ Historically, the U.S. Food and Drug Administration (FDA) has provided some guidance on reporting requirements (including race information).^11^ In 2016, the FDA reexamined race in clinical trials and introduced new, stronger recommendations surrounding race reporting.^11^ While progress is slowly being made the facts remain: enrollment of minorities in oncological trials has stagnated over the past 2 decades.^12^

Recruitment of ethnic minorities is uniquely dependent on trusted community institutions.^13^ Cultural values, patient education level, and beliefs regarding health practices are factors that may conflict with trial recruitment. For this reason, community physicians are often seen as medical “gatekeepers” of trial participation.^14^ Provider knowledge of protocols, cultural competency, communication skills, and personal bias all have direct impact on trial enrollment.^15^ Higher rates of under- and uninsured patients in underrepresented populations also act as barriers to enrollment in trials.^16^ Potential enrollees often see their insurance status as an obstacle to participation.^17^ Moreover, language barriers may also deter enrollment, which is supported by the fact that bilingual providers are more likely to have higher enrollment rates.^18^ Development of community-based initiatives connecting community members to medical providers has demonstrated efficacy in improving participation, though these initiatives have fallen short in recruitment of both minorities and women.^13,17,18^

 Due to their relative epidemiological rarity, brain tumor clinical trials are a particularly important section of oncological trials. In this study, we aimed to investigate the enrollment tendencies and reporting habits of brain tumor clinical trials by analyzing the largest public database of clinical trials: ClinicalTrials.gov. We queried whether race and ethnicity are assessed upon clinical trial enrollment and subsequently reported on clinical trial completion. For those trials reporting race, we then explored their enrollment numbers for major ethnic groups. As a mini case study, we explore the relationship between trial location and racial demographic distributions and socioeconomic status in the 5 boroughs of New York City.

## Materials and Methods

### Data Set

In our analysis, we employed a cross-sectional design strategy for brain tumor trials registered in ClinicalTrials.gov.

### Selection

We selected all interventional trials, registered between July 1, 2005 and November 11, 2017 with a marked “Completed” status, registered with ClinicalTrials.gov using the mesh term “Brain Tumor” and relevant derivative entry terms*. We defined minority groups according to FDA reporting guidelines: Black/AA, White/Caucasian, Hispanic/Latino, Native American/Other (when available).

### Analysis

XML files for all clinical trials were downloaded and queried using a combination of custom scripts, string parsing tools, and command-line packages.^19^ Each trial is assigned a single XML file with complete information including trial title, indications, phase status, etc. All scripts and raw data are available.

### Statistical Analysis

A multivariate logistic regression model was used to assess association between trial characteristics and likelihood to report race using a Wald 95% confidence interval. The model was generated using 6 predictor variables: Phase, Intervention Type, Primary Purpose, Start Year, Enrollment, and Funding source. Using known methods in intervention type classifying, we accounted for trials with multiple interventions by first organizing them into 4 categories: Drug/Biologicals, Procedure/Device, Radiation/Other, Behavioral. For trials with 2 or more categories, trial intervention types were assigned based on existence of the following interventions in strict hierarchical format: 1) Drug/Biologicals 2) Radiation/Other 3) Procedure/Device 4) Behavioral/Genetic. For example, if a trial had 2+ categories, if at least one of those intervention types was a drug, then the entire trial would be labeled as a drug/biological.^20^ A chi-square analysis was done to compare tumor incidence rates against observed enrollment rates with each ethnic

### Obtaining Trial Information

For the trials selected, we obtained results in either of 2 ways. For trials with results on ClinicalTrials.gov, we directly obtained enrollment numbers from the website. For trials without results on ClinicalTrials.gov, we searched PubMed/MEDLINE and Google Scholar for publications related to the trial using the following parameters: trial NCT number, trial authors, trial title, trial descriptors, and author affiliations. In our definition of “Trial results,” we included publications in academic journals, conference proceedings, and abstracts.

## Results

### Overall Race Reporting

**Figure 1.**
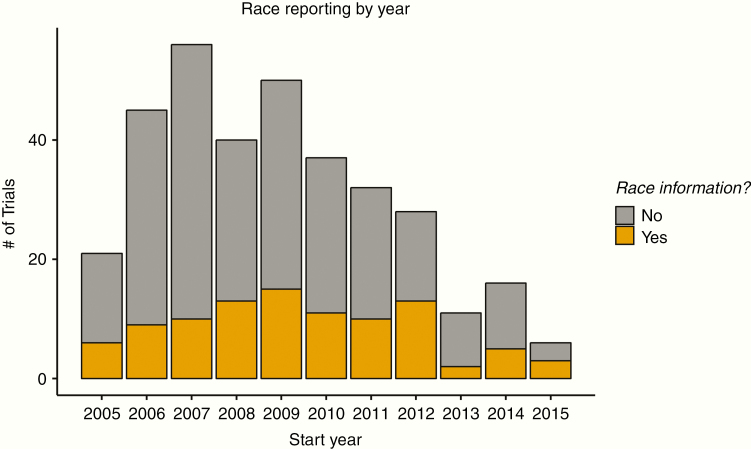
Reporting habits of trials historically sorted by start year of the trial, and that have been completed before November 11, 2017.

In our study, we selected the completed, interventional trials which began after July 1, 2005 and were completed by November 11, 2017. In this time, results of 342 trials were available for North American trials, of which only 97/342 (28.6%) reported race. When stratified by year, trials reporting race varied from 17% to 50%. Trial characteristics are reported in Table 1. In terms of recent trends, rates of reporting for the last 5 years with results (2011–2015) have been 31.2% (10/32) in 2011, 46% (13/28) in 2012, 18% (2/11) in 2013, 31.2% (5/16) in 2014, and 50% (3/6) in 2015. These results are represented graphically in Figure 1.

**Table 1.** Race Reporting Findings for All North American Trials With Results (342), Then Stratified by Intervention Type, Phase Status, and Start Year

	Race reporting?		
	Yes	No	%
North American trials	97	245	28.36%
Intervention type			
Drug	79	212	27.15%
Procedure	10	31	24.39%
Biological	10	26	27.78%
Radiation	9	43	17.31%
Other	22	40	35.48%
Behavioral	5	3	62.50%
Device	2	7	22.22%
Genetic	0	5	0.00%
Phase status			
Phase 1	22	92	19.30%
Phase 1/Phase 2	7	18	28.00%
Phase 2	54	115	31.95%
Phase 3	9	16	36.00%
Phase 4	3	2	60.00%
Start year			
2005	6	15	28.57%
2006	9	36	20.00%
2007	10	46	17.86%
2008	13	27	32.50%
2009	15	35	30.00%
2010	11	26	29.73%
2011	10	22	31.25%
2012	13	15	46.43%
2013	2	9	18.18%
2014	5	11	31.25%
2015	3	3	50.00%

**Figure 2.**
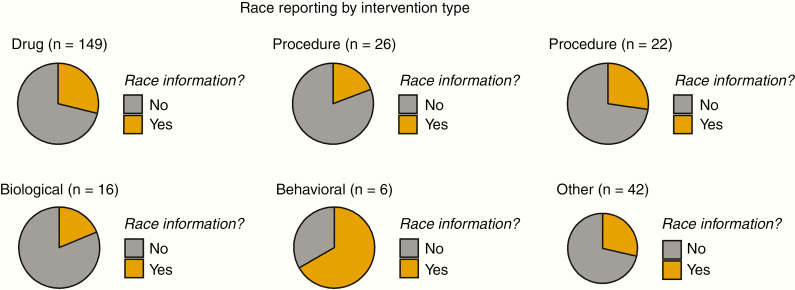
Race reporting trends organized by intervention type.

By intervention type, race reporting varied between 17% and 62%. Only 17% of clinical trials with a radiation intervention type reported race; while as much as 62.5% of behavioral trials reported on race (although these comprised a smaller number of trials overall). Drug/Biological-, procedural-, and radiation-based trials were least likely to report on race. In Figure 2, these rates of reporting race are visualized across intervention types.

**Figure 3.**
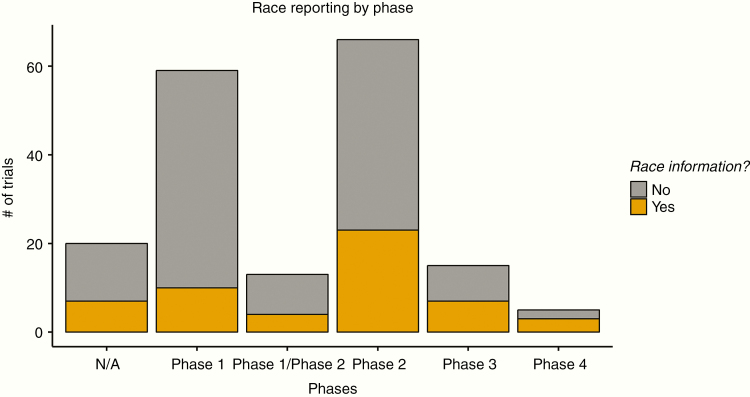
Race reporting habits for trials as a function of phase status.

We also examined trends in race reporting with respect to phase status. The majority of trials with published results were in early phases: 41%, 28%, and 42% in Phase 1, Phase 1/Phase 2, and Phase 2 trials, respectively. Phase 2 trials had higher rates of reporting race than Phase 1 trials (32% vs 19%). Late phase trials (Phase 3 and Phase 4) had higher rates of reporting race—36.0% and 60%, respectively. These trends are shown in Figure 3.

### Trends in Race Enrollment

Aggregated information on racial enrollment was assessed for every trial reporting on race (97/342). Overall, Caucasians had the highest enrollment, comprising 85.3% of all patients during the time period examined. No significant changes in enrollment were observed for the Caucasian group. Blacks, Hispanics, and Asians were the 3 least enrolled racial groups, with enrollment percentages at 4.4%, 3.1%, and 2.3%, respectively. When examined by trial start year, all minority groups saw declining rates of enrollment percentages. Hispanics saw a halving of enrollment percentages from the first time period (2005–2007) to the last (2011–2015). Blacks/AAs also saw a considerable decrease in enrollment during these same time periods. These results are visualized in Table 2.

**Table 2.** Race Composition of Trials Over 3 Time Periods

Race	Years		
	2005–2007	2008–2010	2011–2015
Caucasian	1593/1974 (80.6%)	2120/2429 (87.2%)	2098/2411 (87.0%)
Black/AA	111/1974 (5.6%)	93/2429 (3.8%)	96/2411 (3.9%)
Hispanic	101/1974 (5.1%)	50/2429 (2.1%)	60/2411 (2.5%)
Asian	58/1974 (2.9%)	50/2429 (2.1%)	49/2411 (2.0%)

**Figure 4.**
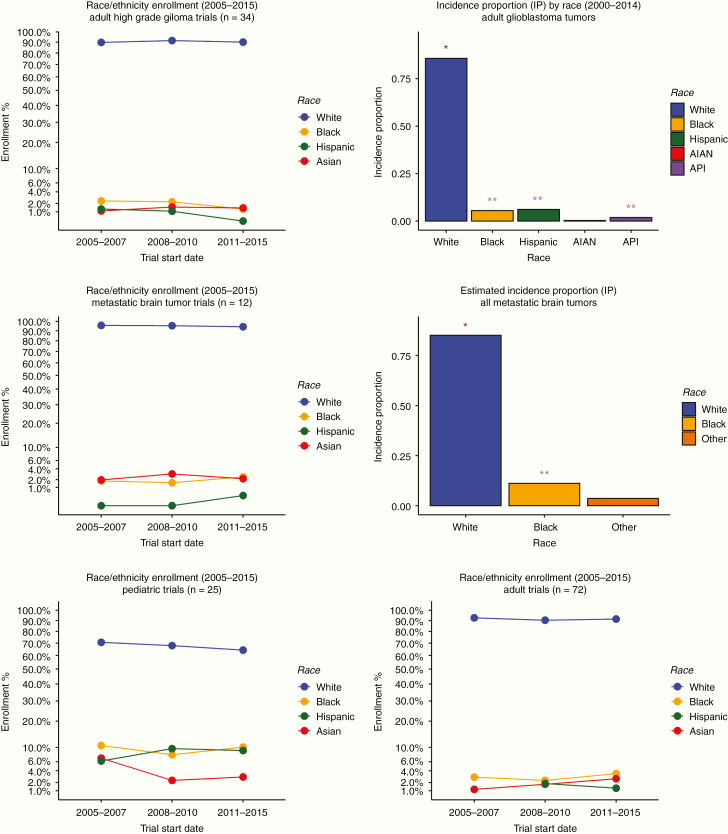
(Top left panel) Enrollment percentages over time for all racial groups analyzed for adult high-grade glioma trials. Single asterisk indicates statistically significant overrepresentation. Double asterisks indicate statistically significant underrepresentation. (Top right panel) Annual age-adjusted incidence rate converted into incidence proportions for all plotted groups, for GBM. Single asterisk indicates statistically significant overrepresentation. Double asterisks indicate statistically significant underrepresentation. (Middle left panel) Enrollment percentages over time for all racial groups analyzed for all metastatic brain tumor trials. (Middle right panel) Annual age-adjusted incidence rate for all plotted groups converted into incidence proportions, for all metastatic tumor types**. (Bottom left panel) Enrollment percentages over time for all racial groups analyzed for all pediatric brain tumor trials. (Bottom right panel) Enrollment percentages over time for all racial groups analyzed for all adult brain tumor trials. *Hispanic AAA IR for all brain tumor types taken from: Ostrom QT, Gittleman H, Xu J, et al. CBTRUS Statistical Report: Primary Brain and Other Central Nervous System Tumors Diagnosed in the United States in 2009–2013. Neuro Oncol. 2016;18(suppl_5):v1–v75. **Estimated from: Davis, Faith G, et al. Toward determining the lifetime occurrence of metastatic brain tumors estimated from 2007 United States cancer incidence data. Neuro-oncology 14.9(2012):1171–1177.

Trial enrollment for minority groups in adult high-grade gliomas was also low. Using the GBM incidence proportion, Whites showed statistically significant overrepresentation in these trials (*P* < .001), while all other minority groups (Asian, Hispanic, Blacks) showed significant underrepresentation. These findings are highlighted in Figure 4A and B.

Trials for metastatic brain tumors were also examined and showed worsening of trial representation and mirrored the same statistical inequalities when comparing estimated incidence proportions with trial enrollment. Whites were statistically overrepresented in trials for metastatic brain tumors (*P* < .001). Blacks were statistically underrepresented in these same trials (*P* < .01).

Subsequently, enrollment in pediatric trials was compared against adult trials. Pediatric trials showed considerable better representation against adult trials for every major race. Whites represented 67% and 91% of pediatric and adult trials, respectively. Blacks comprised 9.5% of all pediatric trials, compared to 2.8% in adult trials. Hispanics represented 8.3% of pediatric trials versus 1.1% in adult trials. Asians made up 5.4% of pediatric trials, versus 1.8% of adult trials.

### A Mini Case Study: New York City

**Figure 5.**
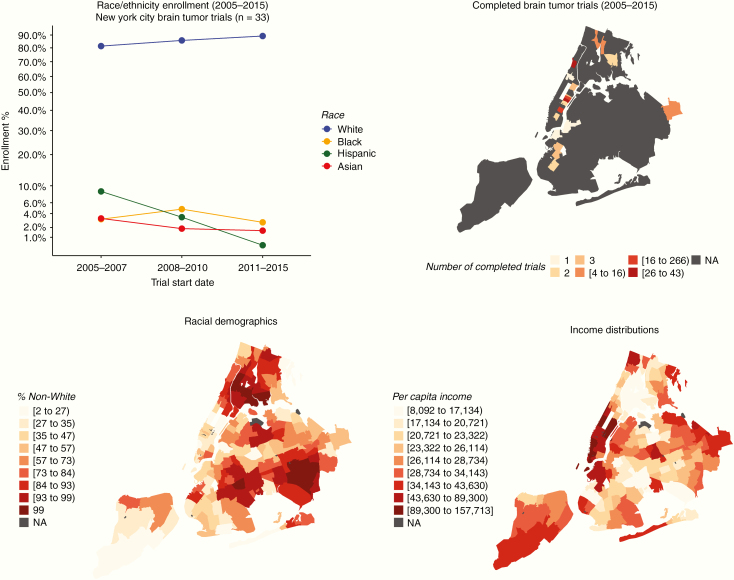
(Top left panel; A) Enrollment percentages over time for all racial groups analyzed for brain tumor trials located within the 5 boroughs of New York City. (Top right panel; B) Density map of completed brain tumor trials for New York City. (Bottom left panel; C) The percentage of non-White minorities in each county within the 5 boroughs. (Bottom right panel; D) Per capita income distribution across the 5 boroughs.

As a case study, we explored the enrollment and recruitment of trials with locations within the 5 boroughs of New York City. Trial locations were sparsely found within the boroughs but were predominantly concentrated in the major academic centers in Manhattan. The most common trial zip code being 10065 (containing Memorial-Sloan Kettering Cancer Center). Trial location zip codes were plotted as densities overlaid on the map of the 5 boroughs in Figure 5B. In Figure 5C, using 2010 U.S. Census Data, racial demographics show a clustering of large non-White populations in areas where, historically, no brain tumor trials have been completed. In order to examine the association of trial location with income, census data on income were obtained and analyzed. In Figure 5D, income distributions show higher per capita incomes follow areas of higher density of clinical trials.

## Discussion

In this analysis, we examined 471 clinical trials across 12 years to discern the trends in race and ancestry reporting and recruitment. We then analyzed the race reporting and enrollment trends of racial and ethnic groups specifically for all trials as well brain tumor trials in North America, which highlighted several important points about the status of clinical trials for brain tumors. First, the majority of brain tumor trials did not report race (>70%), and there is no indication of improvement over time (Figure 1). Second, clinical trials using radiation, drug, and biological interventions were among the least likely to report on race, failing to report on race in the vast majority of cases (>70%). Black/AA, Hispanic, and Asian groups are poorly represented in clinical trials for brain tumors and face declining rates of recruitment. In addition, for many completed trials, results were noticeably absent from the searchable literature. Twenty-seven percent (129/471) of completed trials had no published results.

Despite mounting scientific evidence that treatment response differs by race, only 27% of drug- and biological-based brain tumor clinical trials reported on race. Previous research has shown that subgroup analyses of biological trials have been able to elucidate efficacy when stratifying by minority races even after negative findings in aggregate.^21^ A prime example is the drug BiDil (hydralazine and isosorbide dinitrate), which was initially found to be ineffective in controlling heart failure before being shown by subgroup analysis to be effective in AAs.^21^ Similarly, adequate race reporting in neuro-oncology trials may be able to elucidate differences in response time, survival, and treatment efficacy. Precision medicine via new, low-cost sequencing technologies may add further stratification and provide a high-resolution perspective into differences in treatment response. Similar genetic analysis utilizing precision medicine in clinical trials for lung disease showed that bronchodilator response had lower efficacy in minority children with asthma, which authors were able to attribute to newly discovered genetic risk factors found in these populations.^22^

### Representation in Brain Tumor Trials

In our study, we found that minorities were poorly represented in brain tumor clinical trials. AAs and Hispanics represent 13% and 17% of the population, respectively; however, both were persistently underrepresented throughout the examined time period.^23^ In our analysis, all observed minority groups showed a decreasing enrollment fraction in between the time periods 2008–2010 and 2011–2015 (2.36% from 3.63%). Despite the increasing impact of the globalization of clinical trials and its influence and applicability to diverse populations, these findings underline an already existing problem in the West. Using the incredible diversity of New York City as a model, we showed, despite its tremendous heterogeneity, trial locations are heavily concentrated in high-income, nondiverse areas. These findings highlight an important scientific-cultural alignment that exists between trial composition and neighborhood composition. Interestingly, we also showed that representation in pediatric trials shows greater diversity in enrollment as compared to adult trials.

Potential reasons for the findings detailed fall into several categories. First, only between 8% and 11% of all newly diagnosed GBM patients enter clinical trials.^9^ Those diagnosed require competent and knowledgeable providers to explain a complicated disease process, and require referrals to larger tertiary centers hosting clinical trials. Community providers play a pivotal role in engaging minority groups at the time of diagnosis. Our findings suggest that for community providers, while managing patient populations that are disproportionately affected by major chronic diseases like cardiovascular disease and diabetes, proper diagnosis and referral to appropriate centers for clinical trial recruitment may be a challenge. Moreover, minorities are more likely to carry comorbidities and have poorer management of their disease that may exclude them from pivotal clinical trials.^24,25^ For this reason, strict eligibility criteria may play a quiet role in hindering recruitment. While surrogates for socioeconomic status including education level and average income have a large impact on recruitment, other patient-derived factors like distance from trial location also play a role.^17^ Geographical barriers were cited as one of the largest contributors for trial enrollment refusal.^17^ Accordingly, affluent neighborhoods contained higher overall trial enrollment than those in less affluent neighborhoods.^15^ When researchers attempted to combat this by conducting trials in key urban neighborhoods, accrual rates for minorities were 10 times higher compared to all others.^26^

### Scientific Obligations for Completeness in Reporting

Despite strong verbiage from the FDA and ICMJE, a large amount of unannotated data persists in brain tumor trials—an alarming issue for a field already hampered by the highest failure rates for investigational drugs.^27^ In an area already plagued by high clinical trial failure rates, a catastrophic mortality rate, and low prevalence and incidence, a lack of race reporting further complicates finding patient subgroups where efficacy may occur.

Connecting investigators with community, faith, and legislative leaders in these communities would boost overall as well as equitable enrollment. Additionally, satellite clinics for larger academic centers may provide a direct-to-trial pipeline that eliminates undue travel burdens requiring missing work and additional child care.^26^

The poor recruitment of minorities and lack of information on racial groups in clinical trials is a problem for both current and future treatments, as racial stratification underlies differences in clinical outcomes in oncology and other diseases. Notably, approximately 20% of all new drugs approved between 2008 and 2013 have significant racial differences in metabolism and response rate.^28^ With the NIH Revitalization Act of 1993, trial organizers were mandated to include women and minorities as well as report their enrollment statuses as a strict requirement for funding.^29^ Yet, the data presented here show limited recruitment and a paucity of data on enrollment status.

In addition, we found a trend that researchers in earlier phases were less likely to report on race than in later phase trials. This finding suggests that lower enrollment, smaller resource requirements, and higher failure rates in early phase trials may predispose researchers to exercise less due diligence in proper recruitment of underrepresented minorities. Conversely, larger patient enrollment requirements, higher costs, and greater promise of late phase trials may push researchers to properly recruit and seek diverse patient cohorts, at which point they report on race.

To truly enable an era of “precision medicine” that tailors patients’ treatments to their genetic code, it is requisite to have an understanding of the genetic background of the patients within public databases, significant enough enrollment to quantify how they respond within clinical trials, and accurate reporting for research into efficacy and treatment design to proceed. To our knowledge, no studies have examined either racial reporting or racial representation in brain tumor or neuro-oncology trials, and these results suggest a renewed, widespread effort to rectify this discrepancy is needed.

### Limitations

In our analysis, we used ClinicalTrials.gov to obtain information regarding brain tumor trial status, start date, location, funding status, and phase status. An inherent limitation is that Phase 1 trials, and nondrug trials are currently not required to register on ClinicalTrials.gov. It is likely that there were numerous trials undertaken that were never registered. Although registering on ClinicalTrials.gov is mandated by both the FDA and ICMJE for applicable trials, some data may have been entered incorrectly. In addition, although trials are FDA mandated to update results and completion status on ClinicalTrials.gov, compliance is not always strictly followed.

## Funding

None declared.

*Conflict of interest statement.* The authors declare no existing conflict of interest.

## Authorship statement

Conceptualization: B.T., J.P.G., N.D., C.E.M. Data curation: G.W., B.T. Formal analysis: B.T., B.H. Investigation: B.T., G.W., U.T., B.H. Methodology: B.T., G.W. Resources: B.T., C.E.M., N.D., J.P.G., C.H. Supervision: C.E.M., N.D., J.P.G., C.H. Validation: B.T., G.W., U.T., B.H., C.H. Visualization: B.T. Writing—original draft: B.T., G.W., U.T., B.H. Writing—review & editing: B.T., G.W., U.T., B.H., C.H., J.P.G., C.E.M., N.D.

## References

[1] U.S. Census Bureau Demographic turning points for the United States: population projections for 2020 to 2060. Washington D.C., 2018.

[2] Barnholtz-Sloan JS, Ostrom QT Epidemiology of brain tumors. Neurol Clin NA. 2019;36(3):395–419. 10.1016/j.ncl.2018.04.001 30072062

[3] Ostrom QT, Gittleman H, Stetson L, Virk SM, Barnholtz-Sloan JS Epidemiology of Gliomas. Cancer Treatment and Research. 2015:1-14. 10.1007/978-3-319-12048-5_1 25468222

[4] Simpson JR, Scott CB, Rotman M, et al. Race and prognosis of brain tumor patients entering multicenter clinical trials. A report from the Radiation Therapy Oncology Group. Am J Clin Oncol. 1996;19(2):114–120. 8610632 10.1097/00000421-199604000-00005

[5] Eggly S, Barton E, Winckles A, Penner LA, Albrecht TL A disparity of words: racial differences in oncologist-patient communication about clinical trials. Health Expect. 2015;18(5):1316–1326. 23910630 10.1111/hex.12108 PMC3859820

[6] Kasl RA, Brinson PR, Chambless LB Socioeconomic status does not affect prognosis in patients with glioblastoma multiforme. Surg Neurol Int. 2016;7(suppl 11):S282–S290. 27217966 10.4103/2152-7806.181985 PMC4866060

[7] Pollom EL, Fujimoto DK, Han SS, Harris JP, Tharin SA, Soltys SG Newly diagnosed glioblastoma: adverse socioeconomic factors correlate with delay in radiotherapy initiation and worse overall survival. J Radiat Res. 2018;59(suppl_1):i11–i18. 29432548 10.1093/jrr/rrx103 PMC5868191

[8] Porter AB, Lachance DH, Johnson DR Socioeconomic status and glioblastoma risk: a population-based analysis. Cancer Causes Control. 2015;26(2):179–185. 25421378 10.1007/s10552-014-0496-x

[9] Vanderbeek AM, Rahman R, Fell G, et al. The clinical trials landscape for glioblastoma: is it adequate to develop new treatments? Neuro Oncol. 2018;20(8):1034–1043. 29518210 10.1093/neuonc/noy027 PMC6280141

[10] Cihoric N, Tsikkinis A, Minniti G, et al. Current status and perspectives of interventional clinical trials for glioblastoma - analysis of ClinicalTrials.gov. Radiat Oncol. 2017;12(1):1–12. 28049492 10.1186/s13014-016-0740-5 PMC5210306

[11] US Food and Drug Administration Collection of Race and Ethnicity Data in Clinical Trials: Guidance for Industry and Food and Drug Administration Staff. Rockville, MD 2016.

[12] Kwiatkowski K, Coe K, Bailar JC, Swanson GM Inclusion of minorities and women in cancer clinical trials, a decade later: have we improved? Cancer. 2013;119(16):2956–2963. 23674318 10.1002/cncr.28168

[13] Hamel LM, Penner LA, Albrecht TL, Heath E, Gwede CK, Eggly S Barriers to Clinical Trial Enrollment in Racial and Ethnic Minority Patients With Cancer. Cancer Control. 2016;23(4):327–337. 27842322 10.1177/107327481602300404 PMC5131730

[14] Howerton MW, Gibbons MC, Baffi CR, et al. Provider roles in the recruitment of underrepresented populations to cancer clinical trials. Cancer. 2007;109(3):465–476. 17200964 10.1002/cncr.22436

[15] Noor AM, Sarker D, Vizor S, et al. Effect of patient socioeconomic status on access to early-phase cancer trials. J Clin Oncol. 2013;31(2):224–230. 23213088 10.1200/JCO.2012.45.0999

[16] Sateren BWB, Trimble EL, Abrams J, et al. How sociodemographics, presence of oncology specialists, and hospital cancer programs affect accrual to cancer treatment trials. J Clin Oncol. 2002;20(8): 2109–2117. 11956272 10.1200/JCO.2002.08.056

[17] Lara PN Jr, Higdon R, Lim N, et al. Prospective evaluation of cancer clinical trial accrual patterns: identifying potential barriers to enrollment. J Clin Oncol. 2001;19(6):1728–1733. 11251003 10.1200/JCO.2001.19.6.1728

[18] McCaskill-Stevens W, McKinney MM, Whitman CG, Minasian LM Increasing minority participation in cancer clinical trials: the minority-based community clinical oncology program experience. J Clin Oncol. 2005;23(22):5247–5254. 16051967 10.1200/JCO.2005.22.236

[19] Harold ER. XML: Extensible Markup Language. Wiley; 1998.

[20] Califf RM, Zarin DA, Kramer JM, Sherman RE, Aberle LH Characteristics of clinical trials registered. JAMA. 2012;307(17):1838–1847. 22550198 10.1001/jama.2012.3424 PMC13431740

[21] Temple R, Stockbridge NL. BiDil for Heart Failure in Black Patients: The U.S. Food and Drug Administration Perspective. Annals of Internal Medicine. 2007;146(1):57. 17200223 10.7326/0003-4819-146-1-200701020-00010

[22] Mak ACY, White MJ, Eckalbar WL, et al. Whole-genome sequencing of pharmacogenetic drug response in racially diverse children with asthma. Am J Respir Crit Care Med. 2018;197(12):1552–1564. 29509491 10.1164/rccm.201712-2529OC PMC6006403

[23] U.S. Census Bureau Projections of the Size and Composition of the U.S. Population: 2014 to 2060. Washington D.C, 2015.

[24] Ford JG, Howerton MW, Lai GY, et al. Barriers to recruiting underrepresented populations to cancer clinical trials: a systematic review. Cancer. 2008;112(2):228–242. 18008363 10.1002/cncr.23157

[25] Langford AT, Resnicow K, Dimond EP, Denicoff AM Racial/ethnic differences in clinical trial enrollment, refusal rates, ineligibility, and reasons for decline among patients at sites in the National Cancer Institute’s Community Cancer Centers Program. Cancer. 2014;120(6):877–884. 24327389 10.1002/cncr.28483 PMC3947654

[26] Sharrocks K, Spicer J, Camidge DR, Papa S The impact of socioeconomic status on access to cancer clinical trials. Br J Cancer. 2014;111(9):1684–1687. 25093493 10.1038/bjc.2014.108 PMC4453719

[27] Hay M, Thomas DW, Craighead JL, Economides C, Rosenthal J Clinical development success rates for investigational drugs. Nat Biotechnol. 2014;32(1):40–51. 24406927 10.1038/nbt.2786

[28] McGarry ME, McColley SA Minorities are underrepresented in clinical trials of pharmaceutical agents for cystic fibrosis. Ann Am Thorac Soc. 2016;13(10):1721–1725. 27410177 10.1513/AnnalsATS.201603-192BC PMC5122490

[29] Freedman LS, Simon R, Foulkes MA, et al. Inclusion of women and minorities in clinical trials and the NIH revitalization act of 1993--the perspective of NIH clinical trialists. Control Clin Trials. 1995;16(5):277–285. 8582146 10.1016/0197-2456(95)00048-8

